# Culture Into Perfusion-Assisted Bioreactor Promotes Valve-Like Tissue Maturation of Recellularized Pericardial Membrane

**DOI:** 10.3389/fcvm.2020.00080

**Published:** 2020-05-12

**Authors:** Francesco Amadeo, Marianna Barbuto, Giacomo Bernava, Nicla Savini, Maura Brioschi, Stefano Rizzi, Cristina Banfi, Gianluca Polvani, Maurizio Pesce

**Affiliations:** ^1^Unità di Ingegneria Tissutale Cardiovascolare, Centro Cardiologico Monzino, IRCCS, Milan, Italy; ^2^Unità di Proteomica, Centro Cardiologico Monzino, IRCCS, Milan, Italy; ^3^Dipartimento di Scienze Cliniche e di Comunità, Università degli studi di Milano, Milan, Italy

**Keywords:** biomaterials, valve implant, pericardium, valve interstitial cell, perfusion system

## Abstract

Derivation of tissue-engineered valve replacements is a strategy to overcome the limitations of the current valve prostheses, mechanical, or biological. In an effort to set living pericardial material for aortic valve reconstruction, we have previously assessed the efficiency of a recellularization strategy based on a perfusion system enabling mass transport and homogenous distribution of aortic valve-derived “interstitial” cells inside decellularized pericardial material. In the present report, we show that alternate perfusion promoted a rapid growth of valve cells inside the pericardial material and the activity of a proliferation-supporting pathway, likely controlled by the YAP transcription factor, a crucial component of the Hippo-dependent signaling cascade, especially between 3 and 14 days of culture. Quantitative mass spectrometry analysis of protein content in the tissue constructs showed deposition of valve proteins in the decellularized pericardium with a high variability at day 14 and a reproducible tissue maturation at 21 days. These results represent a step forward in the definition of strategies to produce a fully engineered tissue for replacing the calcified leaflets of failing aortic valves.

## Introduction

Diseased and dysfunctional heart valves are routinely replaced by surgical intervention. About 300,000 heart valve procedures are performed annually worldwide; this number is expected to triple by 2050 with the majority of the patients over the age of 65 (1, 2). Despite their non-modifiable mechanical performance, commercially available mechanical prostheses are prone to thromboembolic complications causing patients to require lifelong anti-coagulation therapy. Biological valves, produced with animal-derived pericardium and valves, or deriving from tissue homografts, undergo structural deterioration, and this is still the principal cause of their failure in the mid/long term. This problem affects significant proportions of patients, especially the young, which mandates re-intervention (3). Deterioration of the biological implants is caused primarily by chronic inflammatory reaction due to the failure to detoxify the fixative remnants in the tissue (4, 5), and/or the incomplete removal of major xeno-antigens (6–10) (α-Gal). A current solution to circumvent these problems consists of the design of tissue engineered heart valves (TEHVs) by combining biodegradable scaffolds and cells of various origins (11, 12). Despite several materials and cell types have been proposed, there are still a number of unresolved problems in TEHVs due to insufficient structural stability of the engineered leaflets and consequent leaflet “retraction” and “thickening” effects (13), which result in TEHVs failure at mid-/long-term (14).

In previous contributions (15, 16), we showed the suitability of a decellularization procedure with ionic/non-ionic detergents to maintain the mechanical properties and reduce the immunogenicity of porcine pericardium. Besides drastically reducing the content of xenoantigens, the treatment also increased the permeability of the tissue, thus making possible the employment of a perfusion bioreactor to enable mass transport through the pericardial matrix and promote stable cellularization (16). Compared to other recellularization techniques to seed cells in valve-competent scaffolds based on static culture (17, 18), the employment of this system enabled a higher penetration of valve interstitial cells (VICs) inside the scaffold and reduced the activation of these cells into myofibroblasts. The aim of the present investigation was to assess the maturation of the “living” pericardium material, particularly concerning the protein deposition inside the matrix and the cellular phenotype at short and long culture time points, in view of scaling up the procedure for clinical transfer.

## Methods

### Cell Culture

Primary aortic VICs were isolated from porcine aortic valve as previously described (15). Cells were cultured up to four passage in Dulbecco's modified Ealge's medium (DMEM, Lonza) containing 1% L-Glutamine (Lonza), 1% penicillin-streptomycin (Lonza), and 10% fetal bovine serum (FBS, Thermo Scientific).

### Decellularization of Porcine Pericardium

Pericardium samples were obtained from four porcine hearts explanted at an authorized slaughterhouse. Decellularization of the tissue was performed according to the optimized protocol described in a previous work (16). Briefly, the pericardium was cut from corresponding left ventricular portion and the adipose tissue was mechanically removed from the surface of the heart. After washing with a sterile solution of phosphate buffered saline (PBS) without Ca^2+^-Mg^2+^ containing Aprotinin (2 μl/mL, Trasylol, Bayer) at 4°C in agitation for 90 min, the tissue was incubated in a hypotonic buffer solution with 10 mM Tris-HCl (pH 8), ethylenediamine tetraacetic acid (0.1%, EDTA), and Aprotinin (2 μl/mL) at 4°C for 16 h under continuous agitation. The tissue was then washed with Milli-Q for 90 min (under agitation at room temperature, RT), and incubated in 1% of Triton X-100 (Sigma) solution at RT for 24 h, followed by extensive rinsing in Milli-Q water for 90 min (RT, in agitation), and incubation in 0.1% sodium dodecyl sulfate (SDS) solution at RT for 24 h. The tissue was finally treated with DNAse (50 U/mL) into a PBS Ca^2+^- Mg^2+^ solution for 90 min at 37°C. Before the culture tissue was sterilized by incubation in BASE.128 (a tissue factory approved sterilization medium) at 4°C for 72 h.

### Dynamic Seeding and Tissue Maturation Through Perfusion Bioreactor

**Figure 1:**
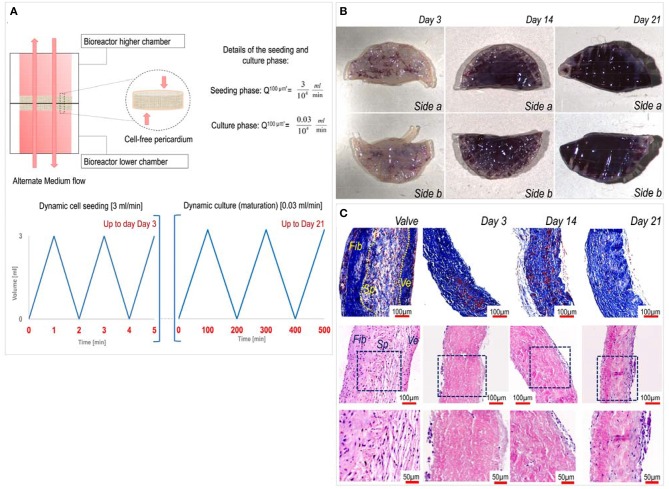
Dynamics of pericardium recellularization process. **(A)** The upper part of the figure describes the alternate motion of the culture medium flow in the chamber of the perfusion bioreactor. The lower part indicates time-dependent representation of the flow during the cell seeding phase (left graph) occurring for the first 3 days and the maturation phase (right graph). **(B)** MTT staining of pericardial patches at different recellularization times shows effective growing of valve cells inside the pericardial matrix up to 21 days of culture. **(C)** Imaging of transversally sectioned recellularized pericardial samples at different times of culture stained with trichrome Masson's (top) and hematoxylin/eosin (bottom). As a comparison, sectioned native aortic valve tissue is included, where a distinction between the three layers, the Fibrosa (Fib), the Spongiosa (Sp), and the Ventricularis (Ve), is shown. Images in the bottom are magnifications of the insets highlighted by blue boxes in the middle panels.

Following the same procedure described by Amadeo et al. (16), decellularized porcine pericardium patches (1 cm diameter) were housed into bioreactor chambers and after that, the bioreactors were assembled. VICs (6 × 10^5^ cells/bioreactor) suspension (10 mL) were dynamically seeded for 3 days applying a high flow at 3 mL/min. After this phase, the recellularized patches were cultured up to 21 days under a low flow rate of 0.03 mL/min (Figure 1A). During the whole process, the alternate flow was applied to enhance nutrient supply and gas exchange. The culture medium was partially (6 mL) changed every 3 days.

### Characterizations of Living Tissue

For the biological characterization of the tissue, three time points were chosen, corresponding to 3, 14, and 21 days of dynamic cell culture. To evaluate the vitality of the cells seeded into the decellularized pericardium, recellularized samples were incubated in 400 μL of 1.2 mM 3-(4,5-dimethylthiazol-2-yl)-2,5-diphenyltetrazolium bromide (MTT) solution at 37°C for 3 h. After that time, images of both sides of the constructs were acquired through Stemi 2000-C Stereo Microscope (Carl Zeiss). For histological analyses, porcine aortic valves and recellularized samples were fixed in 4% paraformaldehyde (4°C overnight) before including in paraffin. Histological sections (5 μm thickness) were cut, dewaxed and stained with Masson's trichrome staining (Bio Optica). Images were acquired using an AxioScope optical microscope (Carl Zeiss). Immufluorescence on recellularized pericardium was performed using antigen retrieval solution (10 mM sodium citrate buffer, 0.05% Tween-20, pH 6) and permeabilization with Triton X-100 (1% v/v) bovine serum albumin (BSA, 3% w/v, Sigma) solution in PBS. Mouse and rabbit anti-Alpha-Smooth Muscle Actin (αSMA), rabbit anti-vimentin, mouse anti-YES-Associated Protein (YAP), and mouse anti-Proliferating Cell Nuclear Antigen (PCNA) were chosen as primary antibodies to evaluate the phenotype of the VICs into the pericardium constructs. The incubation with primary antibodies (αSMA; rabbit, 2 μg/mL, Abcam - αSMA; mouse, 0.7 μg/mL, DAKO - PCNA, mouse, 2 μg/mL, DAKO - YAP, mouse, 2 μg/mL, Santa Cruz Biotechnology, Vimentin, rabbit, 0.45 μg/mL, Cell Signaling) was carried out in a PBS solution containing BSA (3% w/v) overnight at 4°C. After thorough washing with abundant PBS, sections were incubated with 488-donkey anti-mouse (10 μg/mL, Invitrogen) and 594-goat anti-rabbit (10 μg/mL, Invitrogen) secondary antibodies. Cell distribution in the constructs and cells counting were assessed by 4′, 6-diamidino−2-phenylindole (DAPI, 100 μg/mL, Sigma) nuclei staining. All the images were acquired with Apotome fluorescence microscope (Carl Zeiss) or laser scanning confocal microscope (Carl Zeiss).

### Proteomic Assessment

Recellularized samples were snap frozen immediately after unmounting from the culture bioreactors. For proteomic analysis, three independent patches for each time/pericardia samples were stored. Protein extraction was performed for all samples in the same run as follows: homogenization in an extraction buffer containing Tris 0.1 M pH 7.6 and SDS 4% (400 μL of buffer for 50 mg of tissue) performed with the Ultra-Turrax at 24,000 rpm for 30 s on ice, sonication (10 s twice power 6 on ice), and heating at 95°C for 3 min. For label-free quantitative mass spectrometry (LC-MSE), protein extracts were precipitated with the protein precipitation kit (Calbiochem), according to the manufacturer's instructions. Protein pellets were then dissolved in 25 mmol NH_4_HCO_3_ containing 0.1% RapiGest (Waters Corporation), and digested with trypsin as previously described (19). LC-MSE analysis was performed as previously described and analyzed with Progenesis QIP v4.1 (Non-linear dynamics) including normalization of protein abundance considering all the identified proteins and Principal Component Analysis (20, 21). UniProt database (release 2017-6; number of sequence entries for sus scrofa, 3,549) was used for database searches.

### Images and Statistical Analysis

All the data are represented as mean ± standard error. Differences among experimental groups were assessed by GraphPad statistical Software. The type of statistical tests and the number of the replicates included in the analysis are specified in the figure legends.

## Results

As previously shown by us (16), dynamic seeding with an alternate perfusion bioreactor is a promising method to recellularize aldehyde-free decellularized pericardium (see characteristics of the seeding/culture protocol in Figure 1A). In keeping with these findings, porcine VICs penetrated inside the inner part of the decellularized pericardium with a constant increment in MTT color staining on both sides of the patches (Figure 1B), and this preserved tissue structural integrity with possible *de novo* synthesis of extracellular matrix components (Figure 1C). We already showed that a mass spectrometry-based approach is useful to assess the composition of the pericardial matrix before and after decellularization (16). Therefore, here we employed the same technique to get insights on the maturation process of the extracellular matrix in consequence of cells seeding. MS analysis of native porcine valves and recellularized pericardium at different time points rendered a list of 105 proteins (Table S1) that were differentially expressed at different culture times and vs. the native valve tissue. As shown in the table, there were protein groups that were present at specific stages during the maturation of the cellularized tissue in the bioreactor, in addition to one group of proteins that were more abundant in the native valves vs. all the recellularized samples, irrespective of the maturation stage.

**Figure 2:**
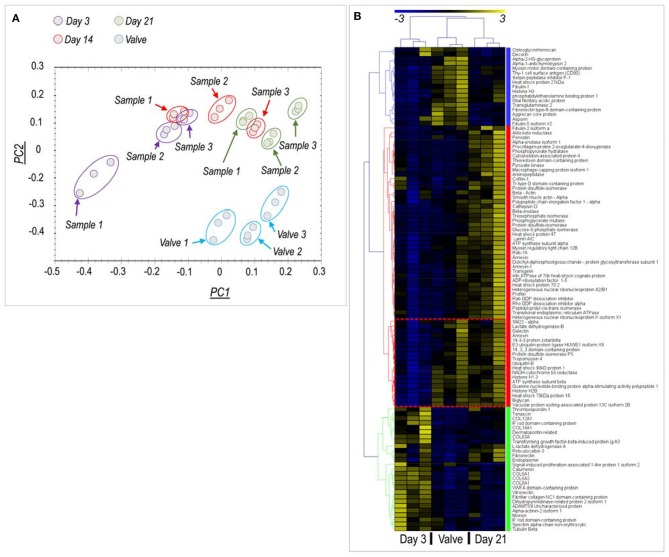
Proteomic analysis of native valves and recellularized pericardial samples. **(A)** Principal component (PC) analysis of protein content in the analyzed samples. Three independent pericardia were recellularized with pig-derived VICs and analyzed at each time point (Samples #1- #3). Each of these samples is represented by experimental triplets evidenced by time-specific color code, for a total of nine samples per time point. Three porcine aortic valves tissues were analyzed in parallel (also in triplets) and are also indicated in the PC analysis. As shown, the alignment of the PC1 recellularized samples at day 21 with the PC1 of the valve tissue reveals a partial restoring of the native protein content by VICs. **(B)** Clusterization analysis of proteins differentially expressed at day 3 and day 21 vs. the native tissue (see Table S1 for description of the proteins and Figure S1 for the analysis including day 14 samples). Three protein clusters (each highlighted by a different color) were identified. A first cluster (characterized by the blue color) contains proteins that were expressed at higher levels in the native valves *vs*. the recellularized samples independently of time. A “red” cluster contained proteins whose expression was relatively higher at day 21 stage than in valve or day 3 samples. Interestingly, a sub-cluster of these proteins (encircled in the heatmap by a red box) were contained at equal levels in day 21 and valve tissues. A “green” cluster containing proteins overrepresented in day 3 recellularized samples was finally found, mainly constituted by structural matrix components. As discussed, this higher abundance might be explained by a lower protein content due to the presence of few cells, which cannot compensate for the protein loss due to decellularization procedure, given the short culture time.

Principal component analysis and clusterization of normalized protein levels Figure 2A indicated a good reproducibility of the recellularization process in the three replicates analyzed for each independently recellularized pericardium samples; in addition, a good data separation was observed between day 3 and day 21 samples, while a partial overlapping of the protein groups representing the day 14 samples and those at the two other culture times was evident. Unsupervised data analysis also showed clusterization of proteins expressed in native valves, day 3 and day 21 samples, and a wider dispersion of data for the day 14 time point (Figure S1).

To analyze better the evolution of the protein content of pericardial samples between the beginning and the end of the culturing period, we eliminated from the analysis the day 14 time-point and performed another data clusterization run, taking into account only the day 3, day 21, and the native valve data. Under these conditions (Figure 2B), proteomic data clearly indicated protein clusters that discriminated each of the three experimental conditions. Interestingly, the number of proteins expressed at the final end point of the culture (day 21) (Red cluster in Figure 2B; *n* = 59 proteins), was higher than the number of proteins more abundant in native valves (Blue cluster in Figure 2B, *n* = 20 proteins) or day 3 recellularization stage (Green cluster in Figure 2B, *n* = 22 proteins) together. In particular, it was noted the presence of a protein sub-cluster in the red group (encircled in Figure 2B) where the protein content was comparable in day 21 recellularized samples and native tissues. This cluster contained, among others, important cellular proteins such as Ubiquitin and Histone H1.3.

**Figure 3:**
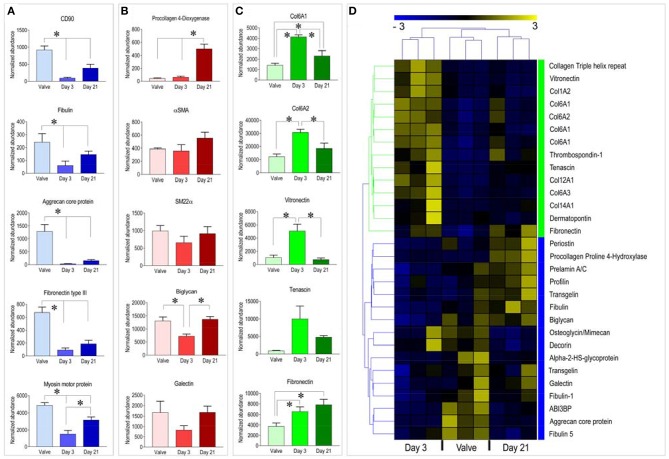
**(A–C)** Example of graphic representation of selected proteins belonging to the three clusters identified by the analysis of proteomic data. Statistical analysis was performed by one way ANOVA with Newman-Keuls *post-hoc* on the normalized abundance of proteins identified by mass-spectrometry (see Table S1). ^*^ represents *P* < 0.05 (*n* = 3). **(D)** Clusterization analysis of proteins with functions in ECM structure/remodeling. Treatment of this data in a separate analysis helped to assess in better details the representation of matrix-related protein distribution during the course of the recellularization procedure. Although separate ECM protein clusters were still identified for the three tissue types, the heatmap clearly shows a higher separation between the day 3 samples and the day 21/native valve tissues, and the higher similarity of the day 21 samples to native valves. This last evidence suggests a trend of the day 21 samples to evolve toward a valve-like tissue.

Analyzing data more in details, and directing our interest on relevant cellular proteins for the valve cells and tissues, we found higher expression of the mesenchymal stem cell marker CD90 (Thy1) and of Fibulin, Aggrecan, and Fibronectin III, three ECM components particularly abundant in the native valve samples compared to the pericardial samples at both culture times. On the other hand, a similar analysis performed on representative proteins of the red cluster (Figure 2B) indicated presence of proteins involved in ECM biogenesis—e.g., Procollagen-Proline-4-Dioxygenase (22), valve maturation—e.g., Biglycan (23), Glycan binding proteins - e.g., Galectin (24) or cellular proteins indicative of the valve-interstitial/smooth muscle cells phenotype (e.g., αSMA and SM22α) at similar levels to the native valves (see also Figure 3B). It was interesting to note that several extracellular structural components such as Collagens, Vitronectin, and Fibronectin were higher represented in recellularized samples at day 3 compared to the other conditions (Figure 3C). For a representation of all the differentially expressed proteins with a function in the extracellular matrix present in the native and recellularized pericardial tissue see Figure 3D, where they clustered into two groups, one evidently more abundant at day 3 of recellularization and mostly composed of ECM structural components (Collagens), and another more represented in native valves and day 21, where matricellular proteins involved in ECM deposition, maturation, remodeling were mostly represented.

**Figure 4:**
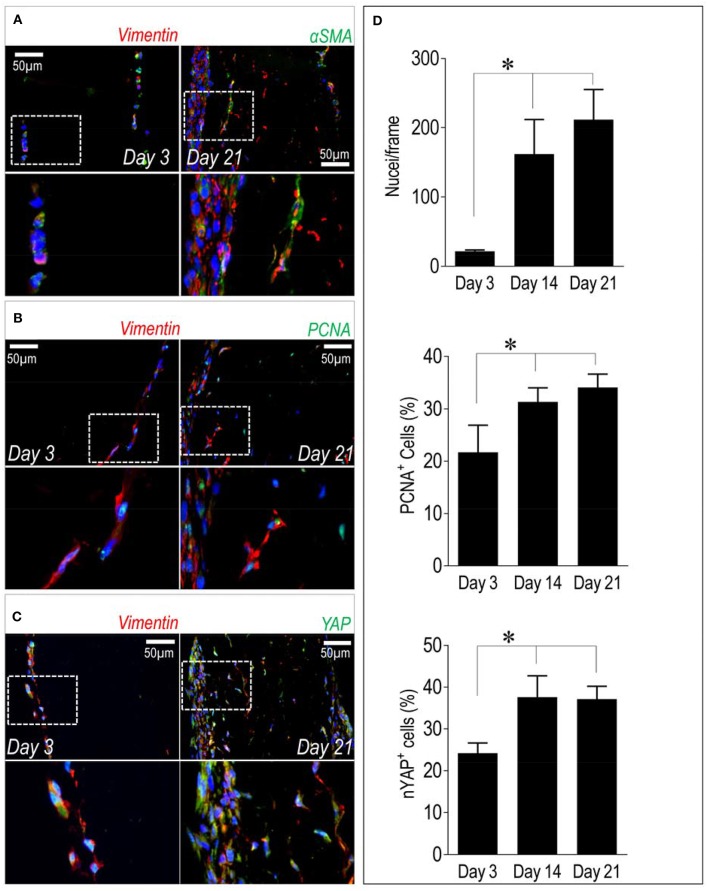
Cellular phenotype and dynamics inside recellularized pericardia. **(A–C)** Immunofluorescence staining showing expression of αSMA, PCNA, and YAP (all in green fluorescence) in conjunction with Vimentin (in red fluorescence) inside the recellularized pericardial tissue at day 3 and 21 of culturing. In each panel the lower micrographs show the magnification of the boxed insets in the higher photographs. **(D)** Quantification of total number of cells by DAPI-stained nuclei counting (upper bar graph), PCNA^+^ cells (middle bar graph) and cells with nuclear localized YAP (nYAP^+^ cells, low bar graph) at all the considered time points. Statistical analysis was performed with one-way ANOVA with Newman Keuls *post-hoc*; ^*^ indicates *P* < 0.05 (*n* = 3).

In a recent publication of our group (25), we have described that stiffness-dependent pathological activation of human valve interstitial cells is related to the activity of the main transcriptional component of the Hippo pathway, the YAP transcription factor (26). Since YAP nuclear translocation in VICs causes increase in proliferation, we determined the number of cells expressing the Ki-67 cell cycle marker and we correlated this with expression of YAP protein in the cells present in our recellularized pericardium samples. In parallel, we determined the expression of αSMA, one of the pathologic markers associated to VICs transformation into myofibroblasts, and Vimentin, a pan-VIC marker (15). Figure 4 shows the result of immunofluorescence staining of the recellularized pericardium at day 3 and day 21 of culture and quantitation of results of cell counting and determination of nuclear YAP (nYAP^+^) and PCNA^+^ cells. As shown in panel A, double immunofluorescence with the pan-VIC marker Vimentin and αSMA antibodies showed a majority of cells stained with Vimentin and a relatively small number of cells expressing also αSMA, especially confined at the surface of the tissue constructs. The double staining including PCNA and YAP in combination with Vimentin (panels B and C), indicated, respectively, a relatively high level of cells with nuclear staining of PCNA (a characteristic of cells in active cell cycle) and expressing YAP at both time points. These observations were corroborated by determination of PCNA^+^ and YAP^+^ cells percentages showing a time-dependent increase between day 3 and day 21 (panel D). Interestingly, while the total number of cells had a trend to increase (although not significantly) from 14 to 21 days, the percentages of PCNA^+^ and YAP^+^ cells were comparable in pericardial samples at day 14 and day 21 of culture.

## Discussion

The quest for novel applications to replace the conventionally used biological material (aldehyde-treated pericardium) and manufacture cardiac valve replacements has prompted a variety of approaches ranging from “off-the-shelf” solutions based on natural/bioartificial materials, to cell seeding into artificially-designed scaffolds manufactured with mechanical characteristics of the natural valves (27). Importantly, despite these advances, the current biological valve implants are still manufactured with animal-derived pericardium treated with aldehydes, a 50-year dated technology with minimal or null modifications introduced since then (28).

Inspired by evidences showing the feasibility of re-engineering entire organs depleted of their original cellular content by recellularization protocols (29), we have endeavored a tissue engineering program with the aim at generating a valve-compliant tissue that may be employed as a living material for aortic valve reconstruction. This solution might be a viable alternative for patients under the age limits for prosthetic valve implantation (30) or, potentially, for correcting congenital valve defects in alternative to conventional procedures (31). The key features that represent a novelty in our procedure compared to others already employed to resolve this problem (18) are that (i) it does not affect mechanical resistance of the pericardial material (15, 32), (ii) it strongly reduces its immunogenic potential by abolishing foreign body reaction (32) and presence of αGAL xenoantigen (15), and (iii) it increases the permeability of the tissue, making it perfusable to achieve an unprecedented efficiency of recellularization (16).

Despite this work is not the first to employ a bioreactor system to assess cell viability inside a decellularized scaffold for cardiac valve engineering (33, 34), it is the first to address the problem of valve maturation of a fully recellularized tissue using a systematic proteomic approach. In fact, our mass spectrometry data clearly showed that compared to the native pericardial membrane, a number of proteins were upregulated in the material as a result of *de-novo* protein synthesis by the introduced valve cells. This occurred with a reproducible time dependence (Figure 2A) and gave rise to distinct proteomic patterns at the beginning and at the end of the culture period (Figure 2B). It was finally interesting to note that 14 days of culture represents a critical time point in the maturation progress of the tissues (Figure 2A, Figure S1), likely due to transitioning of the cells from an active proliferation to a differentiation phase. This clearly establishes a turning point that might be useful to monitoring the quality of the valve tissue generation process based on assessment of tissue construct protein content at this time point.

By analyzing more in details the proteomic data produced by mass spectrometry, we found three protein clusters that were characterized, respectively by, (i) proteins more abundant in the initial culture stage (day 3) compared with the native valve and the final (day 21) pericardium recellularization stage (the “green” cluster in Figure 2B), (ii) proteins whose representation was higher in the native valves, irrespective of the recellularized pericardium culture time (the “blue” protein cluster in Figure 2B) and (iii) proteins whose abundance was higher in day 21 pericardium culture stage compared to day 3 and, at lower extent, to the native valve (the “red cluster” in Figure 2B).

A higher abundance of matrix structural components in decellularized vs. the native samples at short time after cell seeding (the majority in the green protein cluster, Figures 2B, 3A,D) is in line with what already reported for the comparison between the decellularized vs. the native pericardium samples in our previous investigation, where probably it resulted from increase in the relative content of matrix proteins due to removal of the cellular proteins and GAGs (16) and a too low number of cells (Figure 4D). The same reason may account for the higher presence of other components of the ECM such as Tenascin (Figures 2B, 3C,D) at the same time point. It was finally interesting to note that while Tenascin was present in high amount in the native pericardium and it was removed from decellularization process (16), Thrombospondin was transiently expressed by valve cells at the beginning of the recellularization period. Given the general role of Thrombospondins in *pro*-fibrotic activation of VICs (35), it is possible that this factor contributes to active migration of these cells inside the decellularized pericardial matrix.

The larger cluster of proteins differentially expressed in the different conditions contained proteins with higher or equal representation in the recellularized pericardium at day 21 of culture compared to the native valves. It was interesting to find in this group (named the “red” cluster), the presence of enzymes involved in ECM maturation and remodeling such as the Procollagen-Proline 2-Oxoglutarate-4-Dioxygenase (P4HA1) (22) and the Peptidyl-Prolyl cis-trans-Isomerase (PPIase) (36) or Cathepsin-D (37), of enzymes necessary for cell-ECM interactions such as Protein Disulfide Isomerase (PDI) (38), of proteoglycans involved in valve matrix organization such as Biglycan (23), and, finally of cellular proteins typically expressed in valve interstitial cells such as Transgelin/SM22α and αSMA (39). Taken together, these results show that the consistent repopulation of the pericardium with valve cells promotes a valve maturation of the tissue constructs, even if, as showed by the absence other specific valve ECM components such as Aggrecan, Osteoglicyn/Mimecan, Decorin (40) at both stages of recellularized pericardium (the “blue” protein cluster in Figures 2A,C, 3D), suggests that the process was still not complete.

It was also interesting to observe that the number of cells in the tissue constructs tended to reach a plateau in the transition from the 14 to the 21 days of culture (Figure 4). This finding is important for the possibility that cells lose control of their proliferation process once they enter in the pericardial matrix, thus causing risks of uncontrollable growth, and disarrangement of the pericardial matrix. In this respect it has to be noted that the number of cells with nuclear-translocated YAP, a transcription factor connected to the pro-pathologic phenotype in human VICs also reached a plateau (Figure 4) and was lower than in portions of human pathologic aortic valves (25), thereby suggesting an equilibrium between control of proliferation vs. acquisition of valve degeneration markers during valve maturation of the tissue constructs. How this is established it is for now only a matter of speculation, even if, in line with our results on human VICs (25), we hypothesize that this may result from mechanical control of YAP nuclear translocation due to relatively low compliance of the decellularized matrix. Further studies will be done to characterize the local stiffness of the pericardial matrix at different times after recellularization.

In summary, we provide the first example of a partial valve tissue maturation process achieved experimentally by seeding valve cells into a decellularized pericardial matrix. We are now planning new studies with human-derived bone marrow/adipose-derived mesenchymal stem cells and scaled up bioreactors able to efficiently repopulate decellularized pericardial patches with surgical size. It will be also interesting to assess whether mounting the recellularized pericardial material onto stent posts and valve pulse duplicators will promote further biological maturation of the living pericardium material due to mechanical conditioning (41), and will maintain the viability of the cells under tissue mechanical stress (33), as a step forward in translation of the living pericardium material for clinical employment.

## Data Availability Statement

The datasets generated for this study can be found in the Zenodo depository: https://doi.org/10.5281/zenodo.3689596.

## Ethics Statement

Ethical review and approval was not required for the animal study because the porcine material used in the study was obtained at local slaughterhouses.

## Author Contributions

FA, MBa, GB, and NS performed experiments. SR helped in paper revision. MBr and CB analyzed data. GP designed the experiments. MP designed the experiments, analyzed data, and wrote the paper.

## Conflict of Interest

The authors declare that the research was conducted in the absence of any commercial or financial relationships that could be construed as a potential conflict of interest.

## Supplementary Material

Figure S1 Clusterization analysis of proteomic data including samples from all the time points. As shown in the PC analysis (Figure 2A), the protein content in day 14 samples showed a higher variability consistent with day 14 being a critical time point for maturation of the cellularized pericardia samples.

Table S1 List of the differentially expressed proteins revealed by MS. Besides the accession number of each of them it is indicated the result of variance calculation, the fold change, the protein description, and the highest mean condition, colored with the same code adopted for protein clusters represented in Figure 2B.
