# Supplementary figures and images for: Culture Into Perfusion-Assisted Bioreactor Promotes Valve-Like Tissue Maturation of Recellularized Pericardial Membrane
Source: Front Cardiovasc Med. 2020 May 12;7:80. doi: 10.3389/fcvm.2020.00080 (PMC7235194; doi:10.3389/fcvm.2020.00080)

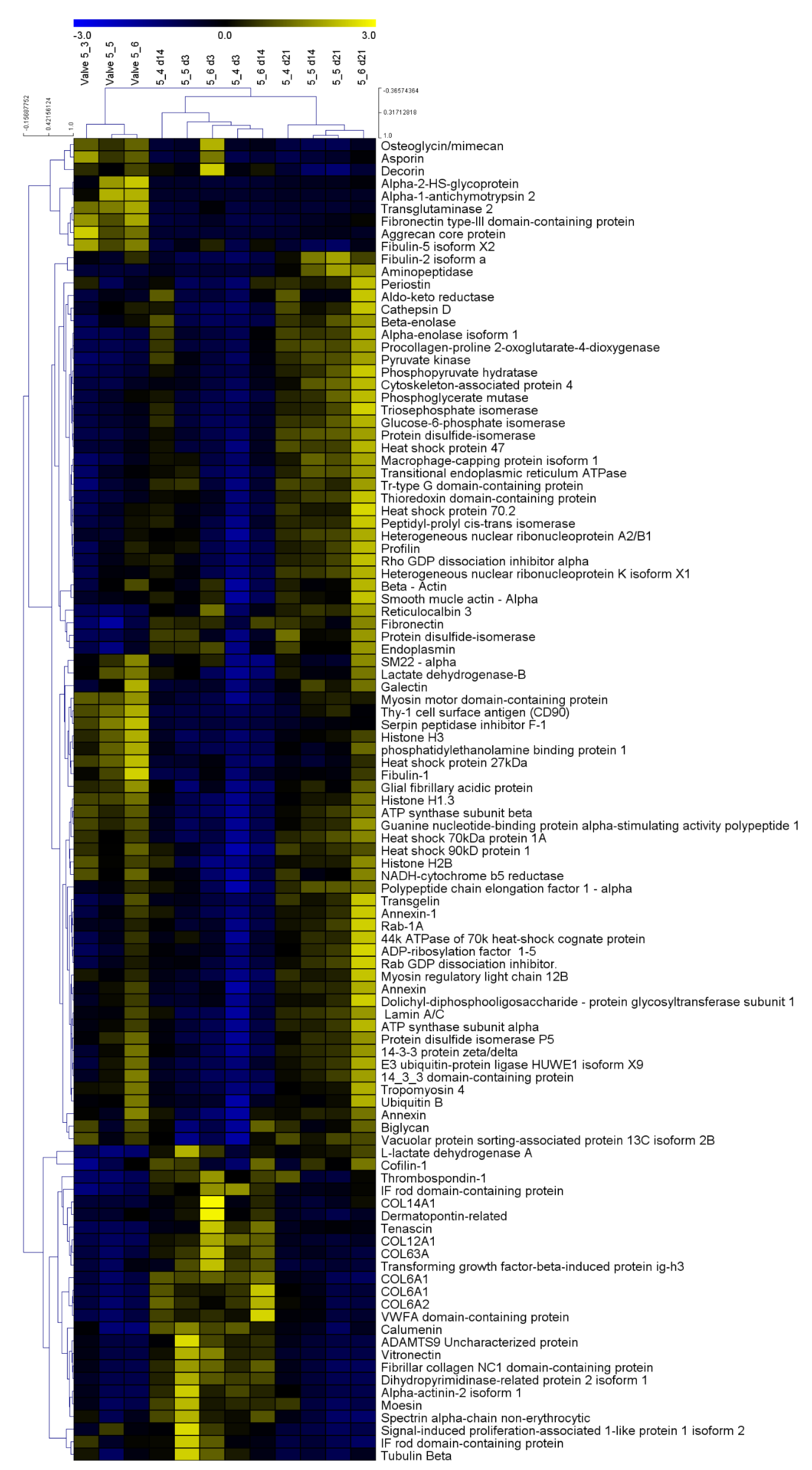

Supplement: Figure S1 — Clusterization analysis of proteomic data including samples from all the time points. As shown in the PC analysis (Figure 2A), the protein content in day 14 samples showed a higher variability consistent with day 14 being a critical time point for maturation of the cellularized pericardia samples. [file Image_1.tif]
