# Supplementary material for: Culture Into Perfusion-Assisted Bioreactor Promotes Valve-Like Tissue Maturation of Recellularized Pericardial Membrane
Source: Front Cardiovasc Med. 2020 May 12;7:80. doi: 10.3389/fcvm.2020.00080 (PMC7235194; doi:10.3389/fcvm.2020.00080)
Supplement: Table S1 — List of the differentially expressed proteins revealed by MS. Besides the accession number of each of them it is indicated the result of variance calculation, the fold change, the protein description, and the highest mean condition, colored with the same code adopted for protein clusters represented in Figure 2B. [file Table_1.docx]

| Accession | Anova (p) | q Value | Max fold change | Power | Highest mean condition | Mass | Description |
| --- | --- | --- | --- | --- | --- | --- | --- |
| *F1SFI7* | 2,20E-08 | 6,13E-08 | 20,47169468 | 1 | valve | 39229,2 | Alpha-2-HS-glycoprotein |
| *F1SDX6* | 2,72E-09 | 1,05E-08 | 14,32559217 | 1 | valve | 78250,84 | Transglutaminase 2 |
| *Q27HS6* | 1,15E-09 | 5,02E-09 | 9,98020156 | 1 | valve | 17456,23 | Thy-1 cell surface antigen (CD90) |
| *F1RVA0* | 1,55E-10 | 7,88E-10 | 3,07091786 | 1 | valve | 15518,17 | Histone H3 |
| *F1SL03* | 1,20E-10 | 6,58E-10 | 7,43108981 | 1 | valve | 104754,7 | Fibronectin type-III domain-containing protein |
| *F1SLA0* | 3,36E-09 | 1,25E-08 | 4,062580004 | 1 | valve | 56335,68 | ATP synthase subunit beta |
| *F1SKR0* | 1,34E-13 | 2,55E-12 | 37,80215526 | 1 | valve | 239513 | Aggrecan core protein |
| *I3L5B3* | 8,39E-08 | 1,97E-07 | 3,220024665 | 1 | valve | 217062,4 | Myosin motor domain-containing protein |
| *Q5IZV0* | 1,57E-08 | 4,70E-08 | 3,103799048 | 1 | valve | 14276,88 | Heat shock protein 27kDa |
| *A0A0H5ANC0* | 9,77E-07 | 1,89E-06 | 3,743755395 | 0,999979 | valve | 34328,51 | Osteoglycin/mimecan |
| *Q53DY6* | 1,56E-09 | 6,41E-09 | 3,560073676 | 1 | valve | 21399,73 | Histone H1.3 |
| *F1RKG8* | 8,99E-11 | 5,37E-10 | 3,946699353 | 1 | valve | 21085,8 | phosphatidylethanolamine binding protein 1 |
| *Q0PM28* | 1,12E-13 | 2,35E-12 | 10,51822726 | 1 | valve | 42578,56 | Serpin peptidase inhibitor F-1 |
| *O62766* | 0,003952715 | 0,0043027 | 1,839745802 | 0,904293 | valve | 22637,91 | SM22α |
| *Q9GMA9* | 1,33E-15 | 3,98E-14 | 104,1410345 | 1 | valve | 46811,66 | Alpha-1-antichymotrypsin 2 |
| *Q29320* | 9,36E-05 | 0,0001279 | 2,159970561 | 0,994985 | valve | 21911,41 | Lactate dehydrogenase-B |
| *F1SUE4* | 4,55E-06 | 7,86E-06 | 3,134339308 | 0,999826 | valve | 42845,73 | Asporin |
| *F1SM61* | 0,000895349 | 0,0010396 | 3,96019596 | 0,965604 | valve | 81907,74 | Fibulin-1 |
| *F1RR02* | 0,001194687 | 0,001357 | 1,734327422 | 0,957426 | valve | 49493,62 | Glial fibrillary acidic protein |
| *F1SD87* | 4,58E-07 | 9,39E-07 | 2,682321666 | 0,999993 | valve | 52503,56 | Fibulin-5 isoform X2 |
| *F1RQI0* | 8,77E-13 | 1,08E-11 | 6,619120766 | 1 | D3 | 102897,7 | COL12A1 |
| *P79272* | 5,27E-09 | 1,78E-08 | 7,096944817 | 1 | D3 | 53162,23 | Vitronectin |
| *F1SMI5* | 4,68E-13 | 6,99E-12 | 10,4812869 | 1 | D3 | 171051,6 | Tenascin |
| *F1SFS3* | 1,36E-06 | 2,56E-06 | 9,348666982 | 0,999966 | D3 | 223453,9 | ADAMTS9 Uncharacterized protein |
| *P79311* | 1,07E-07 | 2,42E-07 | 4,42159284 | 0,999999 | D3 | 129672,9 | Fibrillar collagen NC1 domain-containing protein |
| *I3LJE2* | 3,92E-09 | 1,39E-08 | 4,446769068 | 1 | D3 | 74044,28 | Dihydropyrimidinase-related protein 2 isoform 1 |
| *F1SMN1* | 1,26E-05 | 1,96E-05 | 2,595290364 | 0,999399 | D3 | 37539,37 | Calumenin |
| *Q1T7A5* | 1,08E-10 | 6,08E-10 | 3,604916772 | 1 | D3 | 344103,1 | COL63A |
| *Q1RP81* | 5,47E-09 | 1,82E-08 | 3,693264098 | 1 | D3 | 133537,8 | Thrombospondin-1 |
| *F1RHL9* | 9,07E-07 | 1,77E-06 | 9,168463287 | 0,999981 | D3 | 104302 | Alpha-actinin-2 isoform 1 |
| *Q1T7A8* | 8,03E-10 | 3,65E-09 | 3,055541778 | 1 | D3 | 12723,25 | Type VI collagen alpha-1 chain |
| *F1S285* | 0,000148528 | 0,0001928 | 2,539383861 | 0,992278 | D3 | 193903,2 | COL14A1 |
| *Q29385* | 2,96E-08 | 7,84E-08 | 2,453985547 | 1 | D3 | 36875,8 | L-lactate dehydrogenase A |
| *F1RPV5* | 0,000161917 | 0,0002089 | 2,608923342 | 0,991648 | D3 | 24587,16 | Dermatopontin-related |
| *I3LVS6* | 5,80E-10 | 2,75E-09 | 3,14441949 | 1 | D3 | 56177,64 | IF rod domain-containing protein |
| *F1S3L7* | 2,62E-08 | 7,01E-08 | 5,508968181 | 1 | D3 | 198390,3 | Signal-induced proliferation-associated 1-like protein 1 isoform 2 |
| *F1SQ10* | 0,000305382 | 0,0003754 | 2,138060607 | 0,985438 | D3 | 40243,42 | Decorin |
| *F1RHA7* | 4,75E-08 | 1,24E-07 | 2,503572873 | 1 | D3 | 67332,17 | Transforming growth factor-beta-induced protein ig-h3 |
| *F1SGG2* | 1,58E-07 | 3,46E-07 | 1,976852175 | 0,999999 | D3 | 54427,16 | IF rod domain-containing protein |
| *F1RTN3* | 0,0001368 | 0,0001787 | 1,80667348 | 0,99284 | D3 | 67901,19 | Moesin |
| *F1RR78* | 7,95E-05 | 0,0001093 | 1,656543304 | 0,995717 | D3 | 282962,2 | Spectrin α-chain non-erythrocytic |
| *A5GFX6* | 1,32E-06 | 2,50E-06 | 2,062287716 | 0,999967 | D3 | 50287,35 | Tubulin Beta |
| *Q29568* | 7,71E-11 | 4,74E-10 | 10,21688932 | 1 | D21 | 15866,69 | Phosphopyruvate hydratase |
| *F1RYV6* | 4,25E-07 | 8,84E-07 | 2,616327714 | 0,999994 | D21 | 37060,58 | Aldo-keto reductase |
| *E1CAJ6* | 3,52E-06 | 6,23E-06 | 3,612371374 | 0,999875 | D21 | 48473,62 | Protein disulfide isomerase P5 |
| *Q1W5B8* | 2,20E-11 | 1,64E-10 | 10,57421383 | 1 | D21 | 26895,69 | Triosephosphate isomerase |
| *I3LCN1* | 2,66E-15 | 6,19E-14 | 4,148827952 | 1 | D21 | 38196,53 | Alpha-enolase isoform 1 |
| *F1S8Y5* | 5,46E-08 | 1,36E-07 | 4,483424604 | 1 | D21 | 29415,54 | Phosphoglycerate mutase |
| *F1RFY1* | 0,000402051 | 0,0004885 | 2,405502083 | 0,981681 | D21 | 15214,46 | Profilin |
| *F1SQ01* | 2,92E-05 | 4,32E-05 | 2,326934037 | 0,998464 | D21 | 22137,4 | Thioredoxin domain-containing protein |
| *F1SJB5* | 5,53E-06 | 9,31E-06 | 3,487816273 | 0,999777 | D21 | 39015,53 | Annexin-1 |
| *G9F6X8* | 1,21E-09 | 5,18E-09 | 4,746855076 | 1 | D21 | 56797,78 | Protein disulfide-isomerase |
| *F2Z5U4* | 1,74E-05 | 2,63E-05 | 2,607441887 | 0,999131 | D21 | 22600,54 | Rab-1A |
| *D3K5K1* | 7,10E-10 | 3,30E-09 | 7,736000272 | 1 | D21 | 87717,99 | Periostin |
| *F1RNU9* | 9,12E-12 | 7,62E-11 | 7,320581694 | 1 | D21 | 63134,13 | Glucose-6-phosphate isomerase |
| *A1X898* | 1,67E-15 | 4,35E-14 | 11,29708269 | 1 | D21 | 61277,42 | Procollagen-proline 2-oxoglutarate-4-dioxygenase |
| *F1SPP8* | 3,49E-05 | 5,06E-05 | 5,738412484 | 0,998141 | D21 | 61682,67 | Cytoskeleton-associated protein 4 |
| *Q29578* | 0,006418609 | 0,0068794 | 2,17764971 | 0,871491 | D21 | 71722,99 | 44k ATPase of 70k heat-shock cognate protein |
| *D0G7F7* | 1,43E-06 | 2,68E-06 | 2,57670601 | 0,999963 | D21 | 28635,91 | Tropomyosin 4 |
| *Q4U1U3* | 2,27E-12 | 2,37E-11 | 9,234012233 | 1 | D21 | 44682,47 | Cathepsin D |
| *Q29582* | 7,65E-11 | 4,74E-10 | 3,899241864 | 1 | D21 | 59293,18 | Pyruvate kinase |
| *F1RFY2* | 1,45E-07 | 3,22E-07 | 5,175871992 | 0,999999 | D21 | 47472,31 | Beta-enolase |
| *Q3T929* | 2,48E-08 | 6,74E-08 | 2,529604291 | 1 | D21 | 74460,74 | Lamin A/C |
| *F2Z558* | 1,85E-05 | 2,79E-05 | 2,586349021 | 0,999066 | D21 | 28249,63 | 14-3-3 protein zeta/delta |
| *K7GNJ7* | 3,14E-06 | 5,61E-06 | 6,003484839 | 0,999892 | D21 | 486093,8 | E3 ubiquitin-protein ligase HUWE1 isoform X9 |
| *F1SVB0* | 2,52E-06 | 4,54E-06 | 3,344363999 | 0,99992 | D21 | 39227,25 | Macrophage-capping protein isoform 1 |
| *Q6J267* | 0,001804758 | 0,0020064 | 2,026361969 | 0,942944 | D21 | 15061,91 | Galectin |
| *F1RJ93* | 0,000288873 | 0,0003572 | 3,886149191 | 0,98611 | D21 | 23473,61 | Transgelin |
| *Q9GLE9* | 3,39E-05 | 4,95E-05 | 2,273897856 | 0,9982 | D21 | 83595,48 | Heat shock 90kD protein 1 |
| *F1SIH8* | 0,000594983 | 0,0007065 | 3,044782516 | 0,974887 | D21 | 90115,37 | Transitional endoplasmic reticulum ATPase |
| *D1MAG4* | 5,42E-08 | 1,36E-07 | 3,206923051 | 1 | D21 | 41035,28 | Guanine nucleotide-binding protein alpha-stimulating activity polypeptide 1 |
| *Q8SPK6* | 0,014056565 | 0,0147222 | 1,529624529 | 0,801115 | D21 | 42135,12 | β-Actin |
| *Q9TUP1* | 9,95E-05 | 0,0001351 | 2,37213882 | 0,994683 | D21 | 36063,07 | Heterogeneous nuclear ribonucleoprotein A2/B1 |
| *Q762C2* | 7,54E-05 | 0,0001051 | 2,078199926 | 0,995931 | D21 | 50483,12 | Polypeptide chain elongation factor 1alpha |
| *Q95289* | 0,000406341 | 0,0004909 | 2,432241294 | 0,981521 | D21 | 20643,76 | ADP-ribosylation factor 1-5 |
| *Q9BDE9* | 3,83E-05 | 5,49E-05 | 1,816910562 | 0,997948 | D21 | 70365,96 | Heat shock protein 70.2 |
| *F1RUK8* | 0,000348983 | 0,0004265 | 3,198244276 | 0,983709 | D21 | 50840,13 | Rab GDP dissociation inhibitor, |
| *F1RPS8* | 6,75E-07 | 1,36E-06 | 3,313418555 | 0,999988 | D21 | 57741,24 | ATP synthase subunit alpha |
| *F2Z584;* | 3,35E-09 | 1,25E-08 | 2,306826256 | 1 | D21 | 13936,19 | Histone H2B |
| *Q27HS3* | 0,011653322 | 0,0123007 | 1,53962409 | 0,820047 | D21 | 42408,26 | Smooth mucle actin-α |
| *A5A8V6* | 8,97E-07 | 1,77E-06 | 3,881138556 | 0,999981 | D21 | 70383,53 | Heat shock 70kDa protein 1A |
| *F1SDR7* | 5,42E-07 | 1,10E-06 | 6,514124066 | 0,999991 | D21 | 28226,54 | 14_3_3 domain-containing protein |
| *F1SJQ5* | 8,37E-06 | 1,36E-05 | 2,900938038 | 0,999629 | D21 | 35314,95 | NADH-cytochrome b5 reductase |
| *Q9TTA0* | 3,76E-09 | 1,35E-08 | 3,261414432 | 1 | D21 | 24408,35 | Heat shock protein 47 |
| *E7EI20* | 2,50E-05 | 3,73E-05 | 2,872694365 | 0,998699 | D21 | 23451,44 | Rho GDP dissociation inhibitor alpha |
| *F1SM78* | 1,10E-05 | 1,74E-05 | 3,297204552 | 0,999487 | D21 | 19836,25 | Myosin regulatory light chain 12B |
| *Q29564* | 1,23E-05 | 1,94E-05 | 2,248572514 | 0,999414 | D21 | 18097,5 | Peptidyl-prolyl cis-trans isomerase |
| *F1SPG5* | 3,41E-07 | 7,26E-07 | 5,023190152 | 0,999996 | D21 | 135861 | Fibulin-2 isoform a |
| *F1S073* | 1,66E-08 | 4,83E-08 | 1,738338083 | 1 | D21 | 38864,21 | Annexin |
| *I3LII3* | 0,000180446 | 0,00023 | 2,106799794 | 0,990792 | D21 | 96323,82 | Tr-type G domain-containing protein |
| *F1S0V3* | 0,000239898 | 0,000302 | 2,02329418 | 0,988162 | D21 | 76211,55 | Annexin |
| *E1CAJ5* | 4,96E-05 | 7,01E-05 | 1,930056175 | 0,997323 | D21 | 57315,02 | Protein disulfide-isomerase |
| *F1SPG2* | 0,000642658 | 0,0007588 | 2,81482704 | 0,973322 | D21 | 68796,76 | Dolichyl-diphosphooligosaccharide - protein glycosyltransferase subunit 1 |
| *Q863H9* | 2,23E-08 | 6,13E-08 | 2,079143509 | 1 | D21 | 275810,8 | Fibronectin |
| *F1S2B6* | 1,33E-08 | 4,09E-08 | 1,902959635 | 1 | D21 | 41875,21 | Biglycan |
| *I3LMU6* | 7,41E-06 | 1,22E-05 | 4,470074786 | 0,99968 | D21 | 37625,31 | Reticulocalbin 3 |
| *F1DFL7* | 6,84E-13 | 8,93E-12 | 234,1859242 | 1 | D21 | 109229,7 | Aminopeptidase |
| *A4US66* | 0,00011225 | 0,0001479 | 1,759233912 | 0,994043 | D21 | 23524,11 | Ubiquitin B |
| *I3LQS0* | 0,000211345 | 0,0002677 | 3,660535204 | 0,989404 | D21 | 49542,5 | Heterogeneous nuclear ribonucleoprotein K isoform X1 |
| *F1SRK6* | 0,000759469 | 0,0008868 | 1,46451624 | 0,969644 | D21 | 92690,61 | Endoplasmin |
| *K9IVR2* | 9,61E-12 | 7,72E-11 | 2,03203375 | 1 | D21 | 420106,8 | Vacuolar protein sorting-associated protein 13C isoform 2B |
| *Q1T7A9* | 1,54E-09 | 6,41E-09 | 3,823139476 | 1 | D14 | 10645,81 | COL6A1 |
| *I3LQ84* | 3,61E-09 | 1,32E-08 | 3,193931562 | 1 | D14 | 107887 | COL6A2 |
| *I3LS72* | 5,61E-10 | 2,73E-09 | 4,164893641 | 1 | D14 | 45381,53 | VWFA domain-containing protein |
| *B2CZR7* | 0,00106942 | 0,0012349 | 1,977758802 | 0,960741 | D14 | 10721,09 | Cofilin-1 |

**Table S1. List of the differentially expressed proteins revealed by MS.** Besides the accession number of each of them it is indicated the result of variance calculation, the fold change, the protein description, and the highest mean condition, colored with the same code adopted for protein clusters represented in **Figure 2B**.
